# High-speed spatial frequency domain imaging of rat cortex detects dynamic optical and physiological properties following cardiac arrest and resuscitation

**DOI:** 10.1117/1.NPh.4.4.045008

**Published:** 2017-12-26

**Authors:** Robert H. Wilson, Christian Crouzet, Mohammad Torabzadeh, Afsheen Bazrafkan, Maryam H. Farahabadi, Babak Jamasian, Dishant Donga, Juan Alcocer, Shuhab M. Zaher, Bernard Choi, Yama Akbari, Bruce J. Tromberg

**Affiliations:** a University of California, Beckman Laser Institute, Irvine, California, United States; b University of California, Department of Biomedical Engineering, Irvine, California, United States; c University of California, Department of Neurology, Irvine, California, United States; d University of California, School of Medicine, Irvine, California, United States

**Keywords:** cardiac arrest, brain imaging, cerebral ischemia, hemodynamics, tissue scattering

## Abstract

Quantifying rapidly varying perturbations in cerebral tissue absorption and scattering can potentially help to characterize changes in brain function caused by ischemic trauma. We have developed a platform for rapid intrinsic signal brain optical imaging using macroscopically structured light. The device performs fast, multispectral, spatial frequency domain imaging (SFDI), detecting backscattered light from three-phase binary square-wave projected patterns, which have a much higher refresh rate than sinusoidal patterns used in conventional SFDI. Although not as fast as “single-snapshot” spatial frequency methods that do not require three-phase projection, square-wave patterns allow accurate image demodulation in applications such as small animal imaging where the limited field of view does not allow single-phase demodulation. By using 655, 730, and 850 nm light-emitting diodes, two spatial frequencies ($f_{x}=0$ and $0.3\;{\mathrm{mm}}^{-1}$), three spatial phases (120 deg, 240 deg, and 360 deg), and an overall camera acquisition rate of 167 Hz, we map changes in tissue absorption and reduced scattering parameters ($\mu_{a}$ and $\mu_{s}^{\prime }$) and oxy- and deoxyhemoglobin concentration at ∼14  Hz. We apply this method to a rat model of cardiac arrest (CA) and cardiopulmonary resuscitation (CPR) to quantify hemodynamics and scattering on temporal scales (Δt) ranging from tens of milliseconds to minutes. We observe rapid concurrent spatiotemporal changes in tissue oxygenation and scattering during CA and following CPR, even when the cerebral electrical signal is absent. We conclude that square-wave SFDI provides an effective technical strategy for assessing cortical optical and physiological properties by balancing competing performance demands for fast signal acquisition, small fields of view, and quantitative information content.

## 1 Introduction

Spatial frequency domain imaging (SFDI) has become increasingly used in the field of tissue optics due to its ability to separately quantify absorption and scattering in turbid media over a wide field of view (FOV).^1^^,^^2^ SFDI instrumentation consists of a light source, a projection unit to send a structured light pattern onto the tissue, and a camera to detect the diffusely backscattered light. Performing SFDI at multiple wavelengths can separate the concentrations of tissue chromophores such as oxygenated and deoxygenated hemoglobin.^3^ SFDI is a diffuse optical imaging technique, so its spatial resolution is on the “mesoscopic” length scale (typically on the order of 100  μm), determined by the pixel size of the camera and the optical properties of the incident light and biological tissue. The temporal resolution of SFDI is determined by a number of factors including the frame rate of the camera, the refresh rate of the projector, and the number of spatial frequencies, spatial phases, and wavelengths used. SFDI has been employed in several neuroscience applications, including characterizing stroke,^4^ glioma,^5^ brain injuries,^6^^,^^7^ cortical spreading depression,^8^ evoked stimuli,^9^ Alzheimer’s disease,^10^ and delivery of drugs to glioma tissue^11^ in preclinical models.

SFDI has historically been limited in its ability to resolve fast dynamic changes in cerebral absorption and scattering, due to the constraint that the structured light consists of sinusoidal patterns with three spatial phases for each spatial frequency.^2^ Initial SFDI measurements were acquired with a temporal resolution on the order of ∼1  min due to sequential changing of spatial frequency patterns and phases. Subsequent SFDI instrumentation^1^ generated optical property maps at up to ∼0.2  Hz due to improvements in synchronization between structured light projection and image detection, but this temporal resolution still poses problems for resolving rapid hemodynamics and scattering signal fluctuations in the brain. Diffuse optics methods that rely solely on DC ($f_{x}=0$) light to assess dynamic absorption changes in the brain typically require prior calculation of a differential path-length factor to account for light scattering.^12^^,^^13^

Recently, rapid single-snapshot SFDI methods^14^^–^^19^ for data acquisition and processing have shown potential for overcoming these limitations in temporal resolution. These approaches employ a single fixed frequency projection pattern and Fourier^14^^,^^16^^,^^18^^,^^19^ and/or Hilbert transform^15^^,^^17^^,^^19^ demodulation methods in combination with Monte Carlo-generated lookup tables to calculate optical properties in each pixel. Single-snapshot methods are limited in speed only by signal-to-noise (S/N) and the camera’s acquisition rate. However, they can only be used for relatively large FOV because they need a sufficient number of sinusoidal periods per FOV for accurate image demodulation^15^^,^^17^^,^^18^ and this single snapshot FOV requirement is typically greater than that used for small animal intrinsic signal brain imaging.

To overcome this limitation and maintain high speed and information content, we employ a rapid three-phase square-wave projection technique^17^ and scientific complementary metal-oxide semiconductor (sCMOS) camera, to obtain the first high-speed (Δt≤0.08  s) SFDI images of dynamic optical and physiological properties in the brain. We have previously reported that high-frequency square waves ($f_{x}>0.25\;{\mathrm{mm}}^{-1}$) provide the same information content as sinusoidal patterns due to the fact that tissue functions as a low-pass spatial frequency filter.^17^ Thus, by using binary square-wave patterns with a much higher refresh rate than sinusoids, we can acquire raw SFDI images at an overall frame rate of ∼167  Hz and still take advantage of the high fidelity of the three-phase demodulation approach. The acquisition sequence includes a DC frame ($f_{x}=0$), a frame for each of the three spatial phases (120 deg, 240 deg, and 360 deg) at $f_{x}=0.3\;{\mathrm{mm}}^{-1}$, and three optical wavelengths. As a result, the system generates quantitative tissue optical property maps at 655, 730, and 850 nm with an overall frame rate of ∼14  Hz.

These technical innovations enable rapid spatiotemporal characterization of phenomena related to cardiac arrest (CA)-induced brain injury and cardiopulmonary resuscitation (CPR)-driven cerebral recovery. Real-time measurements are used to separately characterize tissue absorption and scattering changes, enabling visualization of unique contrast features that are difficult or impossible to measure using conventional wide-field intrinsic signal optical imaging. Changes in both tissue absorption and scattering are followed throughout the dynamic periods of CA and post-CPR recovery, even when cerebral electrical signals are absent. This allows for the observation of pulsatile hemodynamics, as well as rapid tissue fluctuations during ischemic and hyperemic phases. Our results support the utility and information content of real-time, square-wave SFDI for assessing cortical optical and physiological properties, particularly in situations where small fields of view and fast, quantitative information content are valuable, such as CA, stroke, traumatic brain injury, and functional activation.

## 2 Methods

### 2.1 Spatial Frequency Domain Imaging

**Fig. 1:**
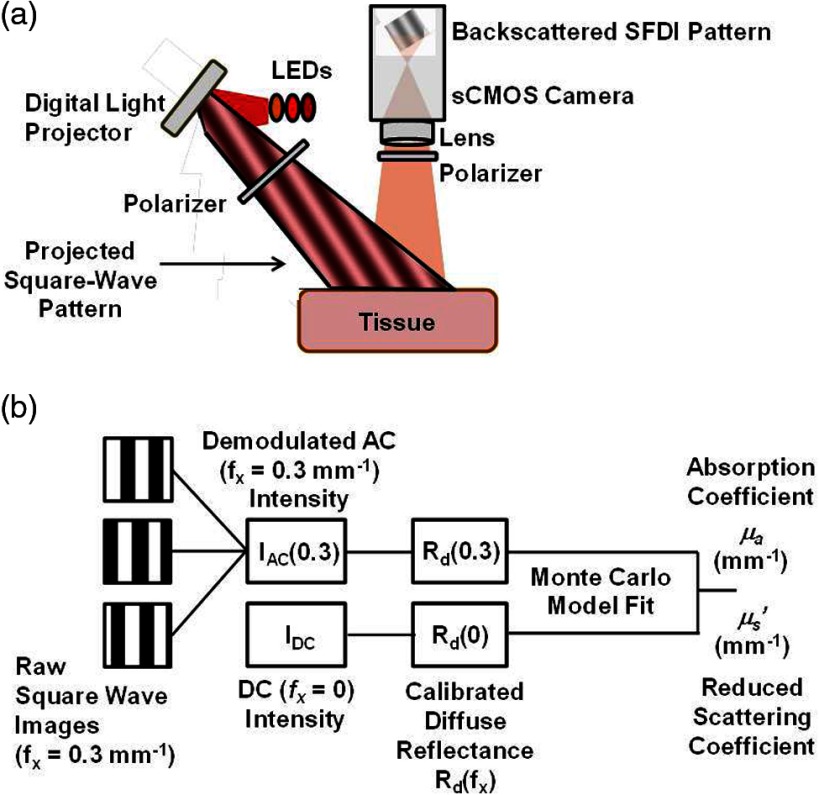
(a) Schematic of rapid SFDI instrumentation. The light source (LEDs of 655, 730, and 850 nm) is coupled to a digital light projector to send square-wave patterns onto the tissue. The patterns are blurred out by the tissue and approximated as sinusoids when they are backscattered from the tissue surface and detected by the sCMOS camera. (b) Workflow of rapid SFDI data processing. Raw images at the three spatial phases are used as inputs into the demodulation algorithm, and the resulting AC intensity, along with the DC ($f_{x}=0$) image, is calibrated with measurements from a tissue-simulating phantom of known optical properties to obtain a map of diffuse reflectance. A Monte Carlo model is fit to the diffuse reflectance values to extract a map of the tissue absorption coefficient $\mu_{a}$ and reduced scattering coefficient $\mu_{s}^{\prime }$ at each wavelength.

The workflow for SFDI has been previously reported by our group.^1^ Briefly, the rapid SFDI setup in this report [Fig. 1(a)] uses three light-emitting diodes (LEDs) as light sources, coupled to a light engine to project spatial frequency patterns onto the tissue. An sCMOS camera detects the backscattered light after it exits the tissue. The camera is synchronized with the light source to serially acquire an image for each spatial pattern and each wavelength.

The light engine (LumiBright™ PR 2910A-100; Innovations in Optics, Woburn, Massachusetts) contains 12 LED bins for visible and near-infrared wavelengths. For this study, two G2 (655 and 230 mW), two H7 (730 nm and 118 mW), and three K1 (850 nm and 62.1 mW) LEDs were used. The sCMOS camera (ORCA-Flash 4.0 V2, Hamamatsu Photonics K.K., Japan) acquired images at 128×128  pixel resolution with a frame rate of 167 Hz. An Arduino Due microcontroller (Sparkfun Electronics, Niwot, Colorado) synchronized the LEDs, camera, and light engine. By running the camera in external edge trigger mode, each exposure was initiated by a transistor-transistor logic (TTL) pulse from the Arduino. The camera then sent a TTL pulse back to the Arduino after the exposure ended. The Arduino used the rising edge of this pulse to externally trigger the LED bank and the DMD, switching serially between the different wavelengths and projection patterns. The frame rate of the camera depends on pixel resolution (128×128), exposure time (1 ms), and running mode (external edge trigger). We also considered delay times to compensate for the rise time of the LEDs and pattern refresh period of the DMD. These delay times and camera parameters provided an overall frame rate of 167 Hz.

To increase imaging speed in our setup, square-wave spatial frequency patterns^17^ are preloaded into the software for the projector. Since these patterns are binary, they can be refreshed more quickly than the sinusoidal patterns typically used in SFDI. This enables image acquisition at a much higher frame rate (167 Hz). The projection and acquisition sequence includes a frame without spatial frequency modulation ($f_{x}=0$), followed by the square-wave pattern ($f_{x}=0.3\;{\mathrm{mm}}^{-1}$) at each of three spatial phases, for each of the three wavelengths. Since the sequence consists of 4×3=12 frames, the optical properties are reconstructed at 167/12∼14  Hz. We previously showed that imaging square waves at a base frequency is the same as effectively the same as imaging a sinusoidal pattern at the same frequency.^17^ This is due to the fact that biological tissue acts as a low pass filter, and the optical power is primarily contained in the lowest (base) frequency component with lesser contribution from higher-frequency harmonics. Thus, there is no expected difference in the imaging depth of our square-wave SFDI method and conventional sinusoidal SFDI.

The SFDI data processing workflow is shown in Fig. 1(b) and has also been described previously by our group.^1^^,^^17^ First, the raw images are demodulated and calibrated against data from a tissue-simulating phantom with known optical properties. In this report, we assume that the square-wave patterns can be treated as sinusoids when they emerge from the tissue due to the diffuse nature of the light propagation and the high spatial frequency of the projected pattern.^17^ Next, the calibrated diffuse reflectance is fit with a Monte Carlo model of photon propagation in tissue to obtain the tissue absorption ($\mu_{a}$) and reduced scattering ($\mu_{s}^{\prime }$) coefficients at each wavelength λ.^1^ The absorption coefficient is fit with the equation $\mu_{a}(\lambda )=2.303({\mathrm{ctHbO}}_{2}\epsilon_{{\mathrm{HbO}}_{2}}+\mathrm{ctHb}\epsilon_{\mathrm{Hb}})$, where $\epsilon_{{\mathrm{HbO}}_{2}}$ and $\epsilon_{\mathrm{Hb}}$ are the molar extinction coefficients of oxygenated and deoxygenated hemoglobin, to extract the concentrations of oxy- and deoxyhemoglobin (${\mathrm{ctHbO}}_{2}$, ctHb) in the brain. The values of ${\mathrm{ctHbO}}_{2}$ and ctHb are used for calculating the cerebral oxygen saturation (${\mathrm{StO}}_{2}$), via the equation ${\mathrm{StO}}_{2}={\mathrm{ctHbO}}_{2}/({\mathrm{ctHbO}}_{2}+\mathrm{ctHb})$). Using this method, $\mu_{a}$ and $\mu_{s}^{\prime }$ were extracted at each individual time point in the experiment.

### 2.2 Animal Model of Cardiac Arrest and Resuscitation

All procedures described in this protocol have been approved by the Institutional Animal Care and Use Committee at the University of California, Irvine (protocol number 2013-3098). Six male Wistar rats of weight ∼300 to 400 g were used for this proof-of-concept study. The details of the animal preparation, cardiac arrest, and CPR have been described previously.^20^ A brief summary of the procedure is as follows. The rat is anesthetized with isoflurane, endotracheally intubated, and connected to a mechanical ventilator (TOPO, Kent Scientific, Torrington, Connecticut) to enable controlled breathing. The femoral artery is cannulated to enable drug delivery, blood sampling, and continuous monitoring of blood pressure via an arterial line.

To provide a window for optical imaging, a craniectomy is performed using a microdrill (Roboz Surgical Instrument Co., Gaithsburg, Maryland) to expose a 4  mm×6  mm region of the skull. Hydration of the exposed region of the brain is maintained by applying saline regularly. The craniectomy was performed on the right hemisphere atop the cortex. For ECoG measurements, two recording electrodes were placed in the front of the brain (2 mm anterior to bregma and 2.5 mm lateral to bregma), and a third recording electrode was placed toward the back of the brain (5.5 mm posterior to bregma and 4 mm left of bregma). A reference electrode was placed in the back of the brain (3 mm posterior to lambda).

To begin the CA portion of the experiment, the isoflurane level is reduced from 2% to 0.5% to 1% while the rat’s inhaled gas is switched from $50\%\;O_{2}+50\%\;N_{2}$ to 100% $O_{2}$. After 2 min, the isoflurane is turned off completely and the rat is given room air (21% $O_{2}$), to enable washout of isoflurane and mimic a typical clinical scenario. This step is necessary to avoid confounding data due to isoflurane since isoflurane can impact CBF and brain function. Concurrent with removal of isoflurane, an intravenous neuromuscular blocking agent (1 mL of 2  mg/kg vecuronium and 1 mL of heparinized saline) is administered for full control of respiratory muscles via the ventilator. After an additional 3 min, the ventilator is then turned off for 5 min. This leads to progressive hypoxic hypercarbic hypotension. We define cardiac arrest as a pulse pressure <10  mmHg and systolic pressure <30  mmHg, after which the rat has reached pulseless electrical activity, a common form of cardiac arrest encountered in the hospital setting.

For all rats in this study, a 5-min asphyxial period was used; this simulated a mild form of cardiac arrest from which the animal was expected to be easily resuscitated. Forty-five seconds before beginning CPR, the ventilator is turned back on with 100% oxygen. Just prior to beginning CPR, 0.01  mg/kg epinephrine, 1  mmol/kg sodium bicarbonate, and 2 mL of heparinized saline are administered intravenously. CPR is performed using manual chest compressions (external cardiac massage) with continuous monitoring of blood pressure to determine when the return of spontaneous circulation (ROSC) is reached. CPR is terminated once ROSC is obtained. Following ROSC, the rat is monitored continuously for an additional ∼2  h. SFDI data are acquired continuously throughout the experiment. Cerebral electrical activity is also monitored continuously over the entire experiment by using electrocorticography (ECoG), as described by our group previously.^20^ At the conclusion of the experiment, the rat is euthanized using pentobarbital.

## 3 Results

**Fig. 2:**
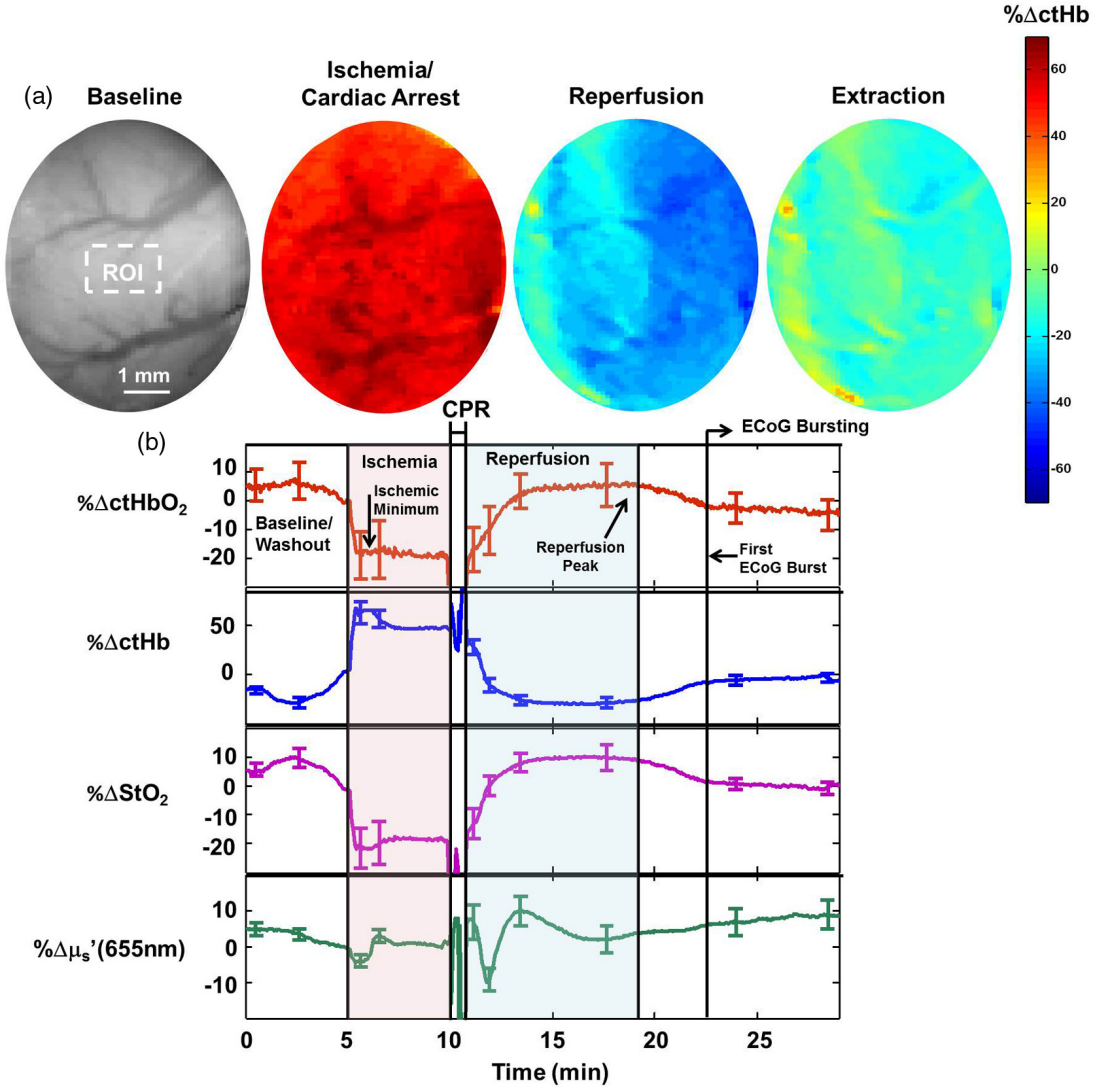
(a) Maps of percentage changes (relative to baseline) in tissue deoxygenated hemoglobin concentration (ctHb) in the brain of a representative rat during asphyxia-induced ischemia and cardiac arrest, post-CPR reperfusion, and extraction of oxygen during resumption of cerebral electrical activity. During asphyxia, the blood supply to the brain is cut off, so ctHb increases sharply due to cerebral metabolism of the remaining oxygen. During post-CPR reperfusion, the brain receives a renewed blood supply, and ctHb decreases significantly because the brain is not yet metabolizing oxygen during this period. During the oxygen extraction phase leading up to resumption of cerebral electrical activity, the brain begins to metabolize oxygen so ctHb increases notably. (b) Percentage changes relative to baseline in tissue deoxyhemoglobin concentration (ctHb, blue), tissue oxyhemoglobin concentration (${\mathrm{ctHbO}}_{2}$, red), tissue oxygen saturation (${\mathrm{StO}}_{2}$, purple), and tissue-reduced scattering coefficient ($\mu_{s}^{\prime }$) at 655 nm (green), over the ROI (dashed white box) shown in Fig. 2(a). Error bars represent the standard deviation over the ROI. The rapid SFDI system provides separate characterization of tissue absorption and scattering with high temporal resolution over the course of the entire experiment. Inflection points in the scattering time-course coincide with CA-related cerebral ischemia, initial reperfusion of the brain following completion of CPR. Curves are shown relative to values just prior to the onset of asphyxia (t∼4.5 to 4.8, rather than t=0) to highlight changes relative to “baseline” levels defined at the end of the anesthesia washout period.

Figure 2(a) shows maps of percentage changes (relative to baseline) in cerebral deoxyhemoglobin concentration (ctHb) for a representative rat during four different phases of the experiment: baseline, ischemia during cardiac arrest, hyperemic reperfusion following CPR, and the oxygen extraction period when initial resumption of ECoG occurs. Figure 2(b) shows time-resolved ${\mathrm{ctHbO}}_{2}$, ctHb, ${\mathrm{StO}}_{2}$, and reduced scattering coefficient ($\mu_{s}^{\prime }$) at 655 nm for this same rat, averaged over the region of interest (ROI) shown in Fig. 2(a). The error bars represent standard deviation over the ROI. We choose to report $\mu_{s}^{\prime }$ at 655 nm because the S/N of the system was best at that wavelength; future studies will incorporate measurements with improved S/N to better elucidate the changes in $\mu_{s}^{\prime }$’ as a function of wavelength. Absolute bulk baseline values (averaged over a larger ROI spanning most of the imaging window) were calculated for ${\mathrm{ctHbO}}_{2}$, ctHb, ${\mathrm{StO}}_{2}$, and $\mu_{s}^{\prime }$ (655 nm) during the end of the washout period (t∼4.5 to 4.8 min) when the effect of anesthesia was at a minimum. These values, reported as mean ± standard deviation over all six rats, were ${\mathrm{ctHbO}}_{2}=85\pm 14\;\mu M$, ctHb=40±6  μM, ${\mathrm{StO}}_{2}=66\pm 3\%$, and $\mu_{s}^{\prime }(655\;\mathrm{nm})=0.87\pm 0.07\;{\mathrm{mm}}^{-1}$. These values, as well as their changes following ischemic insult and neuronal damage, were in a range similar to those reported previously in small-animal brain imaging studies.^4^^,^^21^ The measured baseline ${\mathrm{StO}}_{2}$ was within 9% of that previously measured in the rat brain.^4^^,^^21^ The measured baseline $\mu_{s}^{\prime }$ at 655 nm and the measured ischemic change in ${\mathrm{StO}}_{2}$ were within 10% and 24%, respectively, of the values previously measured by our group.^4^

Figure 2 demonstrates that the instrument detects the expected hemodynamic features of ischemia (sharp decrease in ${\mathrm{StO}}_{2}$ during CA), hyperemic reperfusion (large increase in ${\mathrm{StO}}_{2}$ immediately after CPR), and stabilized hypoperfusion (gradual decrease in ${\mathrm{StO}}_{2}$ following hyperemia, until steady state is reached). By using rapid SFDI, we detect additional dynamic features in the time-evolution of the tissue scattering, separately from the absorption. As seen in Fig. 2(b), these scattering features often exhibit temporal evolution much different from that of the absorption parameters. Thus, Fig. 2 provides a verification of our system and a demonstration of how SFDI disentangles tissue absorption and scattering information at each time point to reveal changes in ${\mathrm{ctHbO}}_{2}$, ctHb, ${\mathrm{StO}}_{2}$, and $\mu_{s}^{\prime }$ that accompany each stage of cerebral response to CA and CPR.

**Fig. 3:**
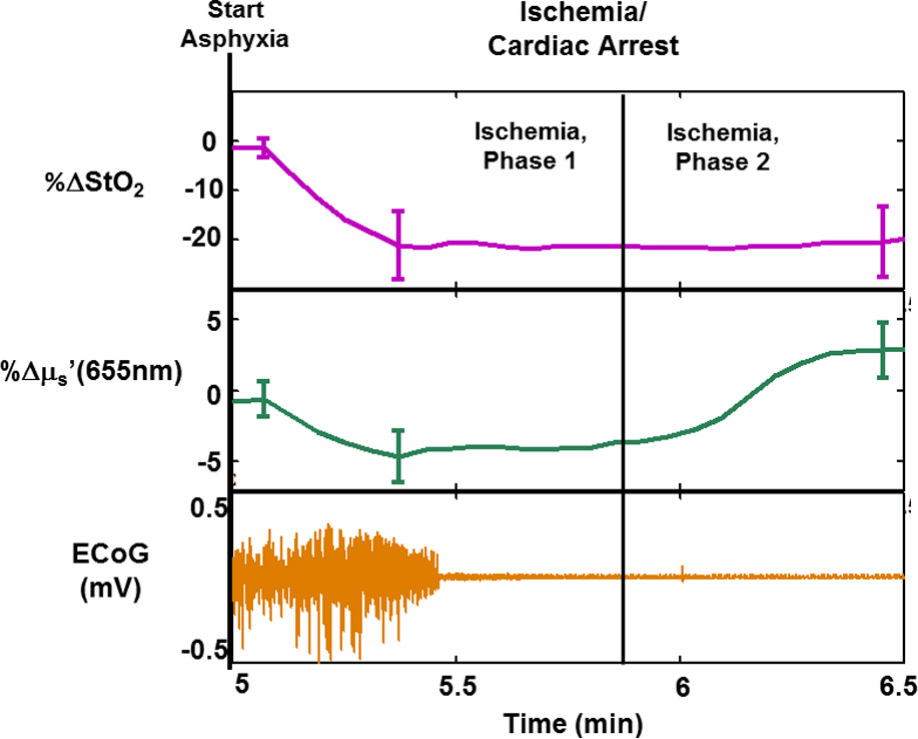
Percentage change relative to baseline in tissue oxygenation (${\mathrm{StO}}_{2}$, top) and reduced scattering coefficient ($\mu_{s}^{\prime }$) at 655 nm (middle), shown atop whole-band ECoG signal (bottom) during cardiac arrest, when ECoG signal goes flat, for the same rat shown in Fig. 2. Error bars represent the standard deviation over the ROI. During the first 45 s of asphyxia (phase I), the loss of electrical activity is accompanied by an almost-immediate sharp decrease of ∼20% in tissue oxygenation and a decrease of ∼5% in tissue scattering. During the second 45 s of asphyxia (phase II), an increase of nearly 10% in tissue scattering is observed well after the ${\mathrm{StO}}_{2}$ has stabilized well below baseline. This scattering change is observed ∼30  s after conventional ECoG has already entered a state of electrocerebral silence.

**Fig. 4:**
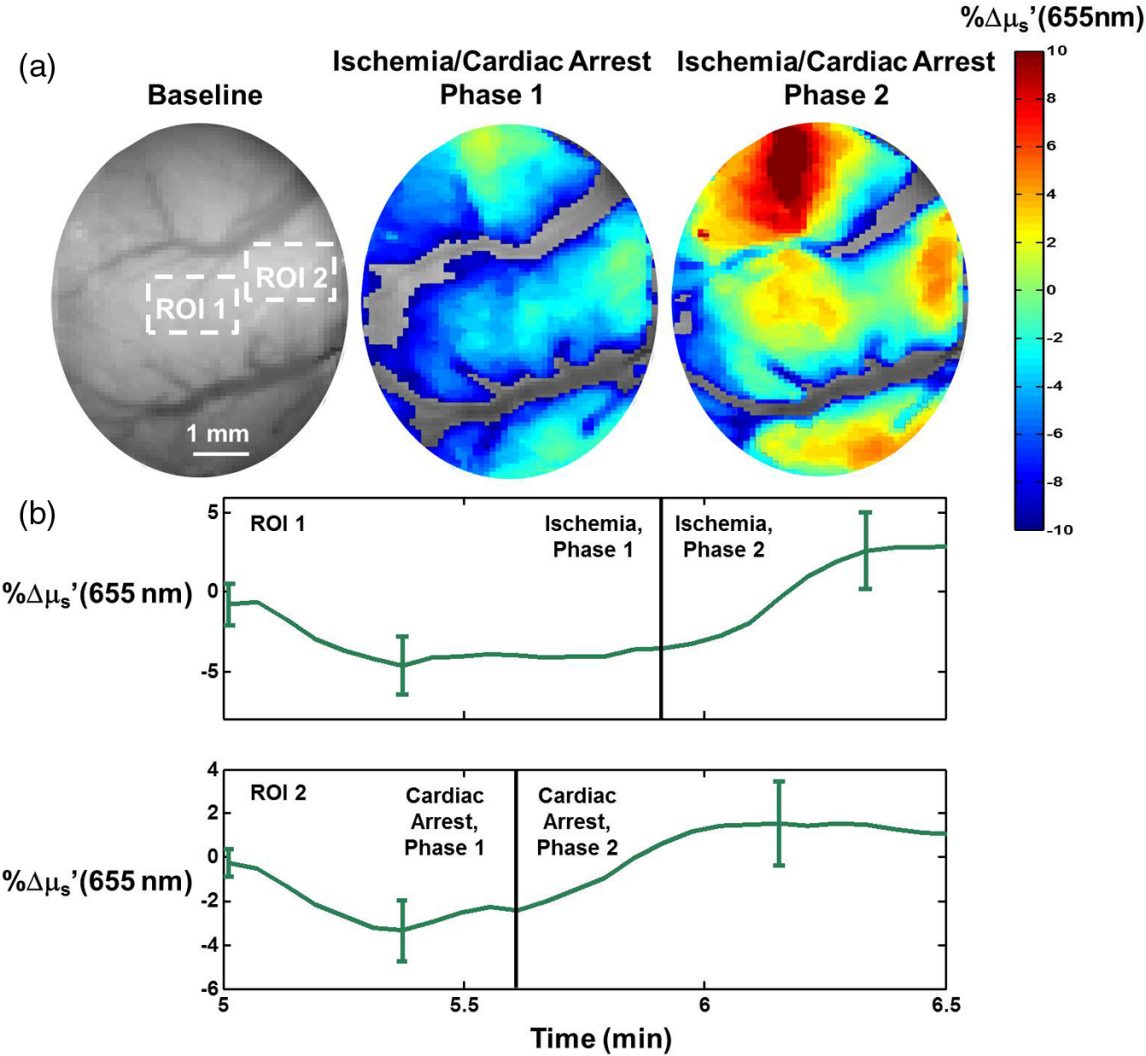
(a) Maps of percentage changes (relative to baseline) in reduced scattering coefficient ($\mu_{s}^{\prime }$) of the brain at 655 nm, during baseline and two different stages of cardiac arrest (CA phase 1 and CA phase 2), for the same rat shown in Fig. 2. These maps demonstrate rapid spatiotemporal variation in scattering in the brain as the animal enters CA. Phase 1 corresponds to the first ∼45  s of ischemia, while phase 2 is representative of the next ∼45  s of that period. Regions devoid of color represent values defined as unphysical ($\%\Delta \mu_{s}^{\prime }>10$ atop major blood vessels). (b) The increase in reduced scattering coefficient at 655 nm during cardiac arrest that denotes transition between the two phases begins at different times for the two different spatial locations (ROI 1 and ROI 2) labeled in the top left image.

**Fig. 5:**
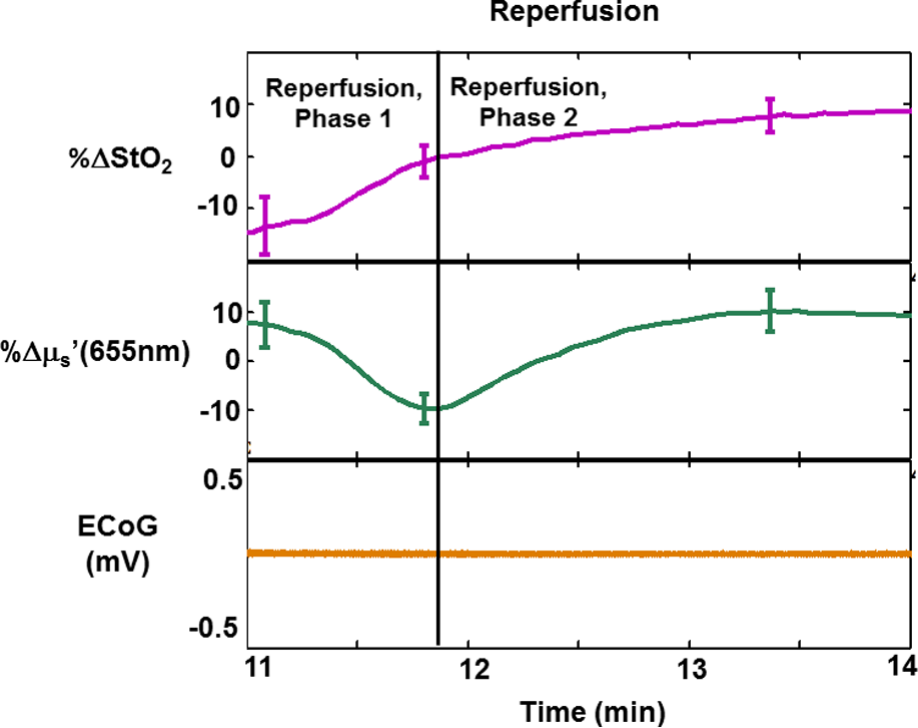
Percentage change relative to baseline in tissue oxygenation (${\mathrm{StO}}_{2}$, top) and reduced scattering coefficient ($\mu_{s}^{\prime }$) at 655 nm (middle), shown atop whole-band ECoG signal (bottom) during the hyperemic reperfusion phase following resuscitation, for the same rat shown in Fig. 2. Error bars represent the standard deviation over the ROI. Even though the ECoG signal is still silent, the ${\mathrm{StO}}_{2}$ and tissue scattering each show unique dynamic behavior. Specifically, there is a gradual overall increase of ∼25% in ${\mathrm{StO}}_{2}$, and a sharp initial decrease of ∼20% (in phase 1), and subsequent recovery (in phase 2), in $\mu_{s}^{\prime }$. This result suggests that SFDI can interrogate previously undetected phenomena during the period immediately following resuscitation, well before changes in electrical activity are seen. The high error bars on the changes in the reduced scattering coefficient are attributable to the high spatiotemporal variation in scattering in the brain during this time period, as shown in Fig. 4.

**Fig. 6:**
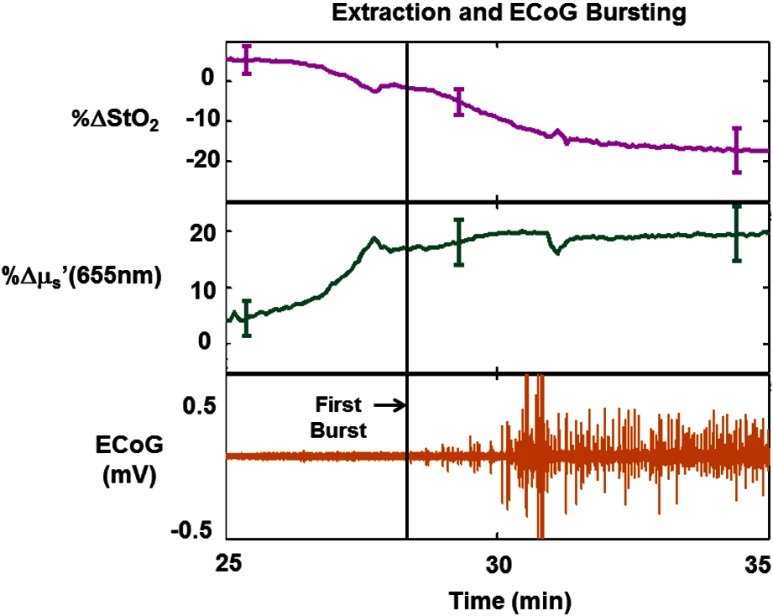
Percentage change relative to baseline in tissue oxygenation (${\mathrm{StO}}_{2}$, top) and reduced scattering coefficient ($\mu_{s}^{\prime }$) at 655 nm (middle), shown atop whole-band ECoG signal (bottom) during the phase where the brain is resuming its extraction of oxygen leading up to initial restoration of ECoG activity (bursting), for a different rat than that shown in Fig. 4. Error bars represent the standard deviation over the ROI. For this rat, the bursting (beginning around t=28  min) coincides with a steady ∼25% overall decrease in tissue oxygenation and a slope change in the time-resolved scattering coefficient. The bursting is preceded by a sharp increase of ∼15% in tissue scattering. This result suggests that SFDI can interrogate rapid changes in tissue structure and function during the resumption of ECoG activity following CPR. However, it is important to note that these sharp scattering changes leading up to ECoG bursting were not seen in every rat in this study.

Figures 3–6 highlight rapid temporal dynamics of tissue oxygenation and scattering [for the same ROI shown in Fig. 2(a)], as well as ECoG, in the three temporal windows labeled in Fig. 2(b): cardiac arrest, post-CPR hyperemic reperfusion, and posthyperemia recovery of cerebral electrical activity. Figure 3 shows a nearly immediate ∼20% decrease in ${\mathrm{StO}}_{2}$ after onset of asphyxia (as ECoG goes flat). Figure 3 also shows that upon onset of asphyxia, there is an initial decrease in $\mu_{s}^{\prime }$ at 655 nm, similar to that reported for time-resolved optical measurements of cerebral infarction.^22^ However, in our study, this initial decrease in $\mu_{s}^{\prime }$ is followed by an increase in $\mu_{s}^{\prime }$ during the period in which the ECoG appears completely flat. The biphasic nature of these temporal scattering dynamics is similar to that reported in a previous study^23^ that used multispectral DC reflectance imaging to examine the effect of anoxia on the rat brain. In Fig. 4(a), spatial variation in the $\mu_{s}^{\prime }$ changes is observed between and within different time windows during the ischemic portion of the experiment. This variation is related to the fact that the $\mu_{s}^{\prime }$ changes are propagating in space as well as time. This phenomenon is further demonstrated in Fig. 4(b), which shows that the changes in $\mu_{s}^{\prime }$ during ischemia occur at different times for different spatial locations on the brain. This spatial propagation was observed in all six rats imaged in this study, and it is similar to that observed in preclinical intrinsic signal optical imaging of cortical spreading depression.^24^^,^^25^ The scattering changes may be related to alterations in neurons^8^ or swelling of mitochondria,^26^ known markers of neuronal injury that have previously been measured optically. Figure 5 shows a sharp ∼20% drop and subsequent rapid recovery of $\mu_{s}^{\prime }$ at 655 nm after resuscitation, as well as a ∼25% increase in ${\mathrm{StO}}_{2}$ after resuscitation. During the time period shown in Fig. 5, there is no observable ECoG signal. Therefore, the dynamic scattering changes and post-CPR increase in cerebral oxygenation appear to occur prior to resumption of cerebral electrical activity.

Figure 6 shows a gradual ∼25% overall drop in ${\mathrm{StO}}_{2}$ and a sharp ∼15% increase in $\mu_{s}^{\prime }$ at 655 nm for a different rat than that used for the previous figures, during resumption of cerebral electrical activity (ECoG “bursting”). This drop in ${\mathrm{StO}}_{2}$ is due to increased extraction of oxygen by the brain, likely caused by resumption of cerebral metabolism, as evidenced by the concurrent increase in ctHb (Fig. 2). The sharp increase in scattering leading up to the initial ECoG burst is shown here to highlight another interesting and novel feature that can be interrogated by rapid SFDI, but it was not observed equally in all of the rats in the study, which is why a different rat was used for Fig. 6 than for the other figures in this report. However, all six rats in this study did undergo an increase in scattering of at least 3% between the minimum value following reperfusion and the maximum value within the 2 min preceding the initial ECoG burst. Overall, the (mean±standard deviation) scattering change during this period was 8.6±5.8%.

**Fig. 7:**
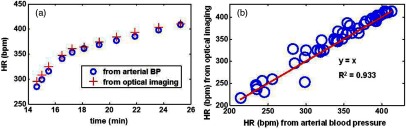
(a) Heart rate for one rat during a 12 min period following resuscitation in a CA/CPR experiment. Values obtained from invasive blood pressure waveform measurement with an arterial line are shown as blue circles, and values obtained from DC intensity at 655 nm are displayed as red crosses. (b) For heart rates below 420 bpm, optically measured heart rate values at multiple different time points for all six rats demonstrate excellent agreement ($R^{2}=0.933$ from fit to line y=x) with values obtained from arterial blood pressure. For heart rates above 420 bpm, the optical heart rate values are unreliable due to inability of the 14 Hz sampling rate to satisfy the Nyquist criterion.

Figure 7 shows that the system, by sampling at 14 Hz, can probe pulsatile variations in the detected optical signal at 655 nm by taking a Fourier transform of the time-resolved data. Figure 7(a) shows good agreement between heart rate extracted from the DC intensity at 655 nm (red crosses) and invasively measured heart rate (blue circles) calculated from blood pressure waveforms acquired with an arterial line during the experiment, for a representative rat. Figure 7(b) shows a scatter plot of heart rate values measured by these two methods (blood pressure-based method on x-axis, optical method on y-axis) acquired at different time points of the experiment for all six rats. Figure 7(b) indicates excellent agreement ($R^{2}=0.933$) to a fit of the line y=x between the optical and arterial-line heart rate values for a sample of values distributed over all six rats in the study, as long as the heart rate was less than 420 bpm. When heart rates above 420 bpm were included in the analysis, poor agreement between the two methods was observed for these higher heart rates. This result is attributed to an inability of the optical sampling rate of ∼14  Hz to fully satisfy the Nyquist sampling criterion for heart rates above 420 bpm, as (14  Hz/2)(60  s/min)=420  bpm. A future study will assess the improvement in extracted heart rate due to modifications to the instrument to increase sampling rate and S/N. Nevertheless, the results shown in Fig. 7 demonstrate that the rapid SFDI platform has the potential to provide quantitative monitoring of the recovery of heart rate following CPR.

The peak changes (mean and standard deviation over all six rats, unless otherwise specified) in ${\mathrm{ctHbO}}_{2}$, ctHb, ${\mathrm{StO}}_{2}$, and $\mu_{s}^{\prime }$ at 655 nm for the different phases of the experiment, as well as the duration of each of these phases, are reported in Tables 1 and 2. These results represent the first (to our knowledge) simultaneous measurement of two-dimensional (2-D) tissue absorption and scattering maps in the brain with temporal resolution of <1  min. These rapid dynamics would be notably more difficult to detect and quantify with the conventional SFDI methods previously reported by our group to image the brain^4^^,^^10^ and other tissues.^27^^,^^28^ It is important to note that in Figs. 2–6, the temporal variations in the scattering appear much different than those in the absorption. This result suggests that these dynamic scattering features are related to real changes in tissue structure and composition, as opposed to artifacts of cross-talk with the changes in tissue absorption.

**Table 1:** Differences (mean±standard deviation) in percentage changes in ${\mathrm{ctHbO}}_{2}$, ctHb, and ${\mathrm{StO}}_{2}$ over three time ranges (as defined in Fig. 2): baseline to ischemic minimum, ischemic minimum to reperfusion peak, and reperfusion peak to first burst, along with time duration of each period. In the middle column, it is important to note that the distribution in reported times from ischemic minimum to hyperemic peak is in part a function of variations in CPR duration.

	Baseline to ischemic minimum	Ischemic minimum to reperfusion peak	Reperfusion peak to first ECoG burst
Difference in $\%\Delta {\mathrm{ctHbO}}_{2}$	−19.4±6.2	32.0±12.8	−9.9±5.9
Difference in %ΔctHb	66.9±19.8	−94.0±14.9	31.0±11.5
Difference in $\%\Delta {\mathrm{StO}}_{2}$	−23.9±7.3	35.8±5.8	−12.0±4.5
Δt (min) between peaks	0.46±0.15	10.4±2.4	7.8±1.8

**Table 2:** Differences (mean±standard deviation) in percentage changes in reduced scattering coefficient ($\mu_{s}^{\prime }$) of the brain at 655 nm over two different time ranges (trough to peak during cardiac arrest and trough to peak during reperfusion, as defined in Fig. 2), along with temporal duration of these two periods. There is a notable difference between the duration of the scattering trough-to-peak period during cardiac arrest and the duration of that period during reperfusion.

	Ischemia*	Reperfusion
Difference in $\%\Delta \mu_{s}^{\prime }$ (655nm) from trough to peak	9.7±4.8	21.2±10.7
Δt (min) from trough to peak	1.1±0.4	2.1±0.4

* Features only observed in five of the six rats.

## 4 Discussion and Conclusions

In this report, we have demonstrated the first (to our knowledge) continuous measurement of 2-D maps of absorption and scattering properties in the brain at frame rates as high as 14 Hz using rapid multispectral SFDI. This technology enables concurrent characterization of dynamic changes in tissue hemoglobin concentration, oxygenation, and scattering in an animal model of cardiac arrest and resuscitation, where changes in tissue structure and function occur on time scales spanning multiple orders of magnitude. We detect changes in tissue optical properties even during periods where there is no observable whole-band cerebral electrical (ECoG) activity in the brain. By imaging at 14 Hz, we can optically monitor the heart rate of the animals throughout the study, as long as it does not exceed the Nyquist limit of ∼420  bpm determined by the image acquisition rate of our system.

During the ischemic period when cardiac arrest is induced, our system characterizes the expected sharp drop in oxygenated hemoglobin concentration and concurrent sharp increase in deoxygenated hemoglobin concentration. This drop is predictably reversed following CPR, where the tissue oxygenation is restored due to reperfusion. These absorption changes are accompanied by biphasic scattering changes (a rapid drop in scattering followed by a rapid increase in scattering) both during cardiac arrest and immediately following CPR. By separating absorption from scattering with high temporal resolution, our rapid SFDI method enables quantitative characterization of these scattering dynamics independently of the absorption dynamics. These scattering changes may be linked to neuronal alterations^8^ and swelling of mitochondria^25^ due to neuronal injury. Future studies will use cellular and molecular assays to assess neuronal structure and function to better understand the underlying mechanisms for the observed tissue scattering changes.

Furthermore, by performing multispectral SFDI measurements concurrently with electroencephalography (ECoG), we observe coupling and uncoupling between tissue optical property changes and ECoG activity. When the brain is initially reperfused following CPR, we detect tissue absorption and scattering changes during a period in which there is no observable whole-band ECoG signal. Following this reperfusion period, we measure a gradual drop in tissue oxygenation (and in some cases, an increase in tissue scattering) leading up to the resumption of ECoG activity (bursting). These findings suggest that multispectral SFDI can detect cerebral extraction of oxygen as the brain resumes its metabolic and electrical activity. Since these absorption and scattering dynamics begin well before ECoG bursting resumes, it is possible that they may be predictive of the initial burst and/or the longer-term recovery of ECoG signal. The predictive value of optical signals for this application was previously reported using laser speckle imaging (LSI),^20^ and a subsequent study will examine potential improvements to the predictive power of LSI for this application when combined with multispectral SFDI.

Several limitations of, and potential improvements to, the rapid SFDI technology will be addressed in subsequent studies. Future work will include rigorous characterization of the limitations of the approximation that square waves can be treated as sinusoids once they emerge from the tissue. Future studies will also investigate whether single snapshot SFDI techniques,^15^^,^^18^^,^^19^ or compressed sensing technology,^29^ can be incorporated into the method reported here to further increase imaging speed. Current single-snapshot methods require an FOV of at least 4 cm to provide enough spatial periods for accurate demodulation. Therefore, the square-wave SFDI technique reported here represents a valuable alternative to single-snapshot methods, because it can be employed with the reduced FOVs used in small-animal brain imaging.

Additional future studies will focus on more detailed analysis of the mechanisms behind the fascinating, and previously elusive, brain tissue-optics phenomena observed in this report. It is possible that next-generation high-speed SFDI technology will be able to establish absorption- and scattering-based metrics to rapidly evaluate the effect of different clinically translatable interventions, performed during CA or immediately following CPR, on longer-term cerebroelectrical outcome.

By acquiring rapid SFDI data directly from the brain, the system reported in this paper provides maps of cerebral absorption and scattering changes with high temporal resolution (∼14  Hz). This enables characterization of rapid changes in tissue scattering properties that may be related to perturbations in structure and function of neurons and mitochondria, in addition to rapid hemodynamic changes due to ischemia and reperfusion. Typical intrinsic signal optical imaging technology cannot resolve these properties independently of tissue absorption with a frame rate on this order of magnitude. Therefore, our technique can potentially provide new quantitative insights into cerebral hemodynamics and metabolic changes during a wide range of dynamic ischemic processes such as CA/CPR, stroke, seizure, and traumatic brain injury.
